# Supplementary material for: Induction of hepatitis B core protein aggregation targeting an unconventional binding site
Source: eLife. 2025 Mar 26;13:RP98827. doi: 10.7554/eLife.98827 (PMC11942178; doi:10.7554/eLife.98827)
Supplement: Supplementary file 1. — ITC200 instrument’s specifications used for the interaction analysis between peptides and capsids. [file elife-98827-supp1.docx]

| *Number of injections* | 14-16 |  |  |
| --- | --- | --- | --- |
| *Cell Temperature (°C)* | 20 |  |  |
| *Reference power (μCal/s)* | 11 |  |  |
| *Initial delay (s)* | 60 -180 | **Injection parameters:** |  |
| *Syringe concentration (mM)* | 0.1 – 1.5 for peptide dimers  1.6 – 2 mM for DM  4 mM for geraniol  2-3 mM for the geraniol dimer | **Volume (μL)** | 2.5 – 2.8 |
| *Cell concentration (mM)* | 0.025 - 0.15 HBc | **duration (s)** | 5 – 5.6 |
| *Stirring speed (rpm)* | 600 | **Spacing (s)** | 180/240 |
| *Feedback mode* | high | **Filter period (s)** | 5/1 |
| *First injection* | No first injection | **Cell volume (mL)** | 0.200 |
