# Supplementary material for: Induction of hepatitis B core protein aggregation targeting an unconventional binding site
Source: eLife. 2025 Mar 26;13:RP98827. doi: 10.7554/eLife.98827 (PMC11942178; doi:10.7554/eLife.98827)
Supplement: Supplementary file 2. — Summary of thermodynamic parameters obtained by ITC experiments using the peptide dimers and fl wt HBc capsids. In case of the P2 dimer, the deviations represent deviations of fit since only one ITC experiment was performed. N represents stoichiometry. [file elife-98827-supp2.docx]

| Peptide dimer | N | K_a_ $\frac{\mathbf{1}}{\mathbf{M}}$ | K_D_ µM | ∆H $\frac{\mathbf{cal}}{\mathbf{mol}}$ | ∆S $\frac{\mathbf{cal}}{\boldsymbol{mol\times K}}$ | Repeats |
| --- | --- | --- | --- | --- | --- | --- |
| **SLLGRM dimer (4)** | 0.15 ±0.02 | 210000 ±30000 | 4.9 ±0.7 | -25 ±3 | -62 ±10 | 5 |
| **P2 dimer (5)** | 0.157 ±0.014 | 500000 ±110000 | 1.9 ±0.4 | -30 ±3 | -76 ±n/a | 1 |
| **P1 dimer (6)** | 0.19 ±0.04 | 6000000 ±6000000 | 0.3 ±0.2 | -26 ±4 | -58 ±15 | 4 |
| **P1dC (7)** | 0.196 ±0.005 | 2490000 ±230000 | 0.42 ±0.04 | -21.7 ±0.6 | -45 ±2 | 3 |
| **DM (1)** | 1.1 ±0.1 | 8000 +/-2300 | 133 ±38 | -2.5 ±0.7 | 9.2 ±2.6 | 3 |
| **Geraniol (2)** | 1.01±0.036 | 10600+/-900 | 94.3 ±7.9 | -4.3 ±0.2 | 3.9 ±n/a | 1 |
| **Geranyl dimer (3)** | 0.236±0.037 | 16000+/-2000 | 62.5 ±7.9 | -14.4 ±2.6 | -30 ±n/a | 1 |
