# Supplementary material for: Induction of hepatitis B core protein aggregation targeting an unconventional binding site
Source: eLife. 2025 Mar 26;13:RP98827. doi: 10.7554/eLife.98827 (PMC11942178; doi:10.7554/eLife.98827)
Supplement: Supplementary file 3. — Obtained raw grayscale values from P1 full positional scan in µSPOT format. Each box corresponds to a single-point variation of the P1 peptide sequence (horizontal) as indicated in the first column. The raw intensity values presented here were used for calculating the fold intensity change of each point variation against the wildtype sequence. Data are presented as mean of n=3 microarray slides with SD. [file elife-98827-supp3.docx]

|  | **M** | **H** | **R** | **S** | **L** | **L** | **G** | **R** | **M** | **K** | **G** | **A** |
| --- | --- | --- | --- | --- | --- | --- | --- | --- | --- | --- | --- | --- |
| **A** | 28.29±8.01 | 29.76±2.22 | 98.61±16.96 | 10.15±3.11 | 8.31±4.76 | 5.55±2.14 | 5.9±0.71 | 55.58±22.38 | 11.72±0.47 | 57.66±14.23 | 16.98±4.54 | 24.35±6.34 |
| **C** | 19.43±7.82 | 22.13±2.95 | 34.85±8.45 | 4.47±1.85 | 6.67±3.89 | 4.78±1.94 | 4.24±1.06 | 7.42±4.58 | 4.5±1.31 | 15.67±8.52 | 7.61±3.77 | 8.92±3.67 |
| **D** | 41.36±8.62 | 40.58±9.04 | 121.37±4.42 | 13.66±1.88 | 5.77±3.05 | 4.61±2.27 | 4.92±1.9 | 11.37±3.38 | 3.94±1.44 | 42.48±12.79 | 33.5±8.31 | 15.97±5.07 |
| **E** | 27.72±9.75 | 74.46±26.92 | 174.74±10.87 | 22.15±6.15 | 5.87±2.73 | 5.12±2.83 | 5.19±1.01 | 125.85±26.07 | 12.24±4.47 | 214.54±10.27 | 69.7±11.56 | 20.01±4.57 |
| **F** | 20.39±7.92 | 30.83±6.54 | 82.39±11.3 | 18.17±1.59 | 5.37±2.17 | 4.32±2.34 | 5.01±1.13 | 71.47±10.55 | 97.92±44.3 | 36.67±6.8 | 19.25±4.34 | 8.11±1.93 |
| **G** | 23.03±7.29 | 31.38±5.87 | 74.75±12.89 | 17.14±2.46 | 4.93±1.54 | 4.03±2.56 | 24.35±6.34 | 14.7±2.45 | 6.91±2.98 | 25.3±4.33 | 24.35±6.34 | 11.49±3.45 |
| **H** | 21.14±7.86 | 24.35±6.34 | 59.98±9.75 | 18.74±4.3 | 7.24±1.91 | 3.47±1.98 | 4.85±2.52 | 11.25±3.78 | 10.4±2.62 | 29.27±9.36 | 15.37±4.17 | 14.05±3.24 |
| **I** | 20.6±5.3 | 29.76±6.65 | 53.52±3.88 | 13.52±7.8 | 17.48±1 | 3.5±1.82 | 4.4±2.08 | 58.16±15.17 | 131.06±32.41 | 160.1±29.36 | 45.69±10.05 | 21.72±4.15 |
| **K** | 19.65±8.04 | 21.14±3.01 | 36.94±6.51 | 10.49±4.75 | 11.69±1.09 | 2.88±1.77 | 4.32±2.52 | 14.46±4.98 | 13.67±3.91 | 24.35±6.34 | 16.66±2.36 | 8.21±1.39 |
| **L** | 19.9±4.19 | 37.55±5.86 | 46.32±6.64 | 9.58±3.9 | 24.35±6.34 | 24.35±6.34 | 4.13±2.54 | 17.18±6.39 | 112.4±19.28 | 48.01±12.59 | 48.89±11.32 | 15.77±1.8 |
| **M** | 24.35±6.34 | 38.05±1.54 | 88.79±10.41 | 11.34±2.2 | 6.32±4.08 | 2.28±1.77 | 3.93±2.27 | 38.17±15.55 | 24.35±6.34 | 45.9±11.89 | 53.16±13.45 | 13.2±0.38 |
| **N** | 23.46±4.29 | 32.64±10.78 | 60.64±4.04 | 9.95±2.75 | 4.77±2.71 | 3.21±1.78 | 7.19±2.82 | 12.72±6.84 | 8.52±1.93 | 23.98±7.23 | 24.2±9.29 | 14.78±1.73 |
| **P** | 25±4.8 | 30.7±7 | 53.35±12.52 | 15.29±3.14 | 4.99±2.49 | 3.64±2.19 | 5.33±2.27 | 13.43±9.39 | 7.96±1.94 | 7.21±2.41 | 14.45±7.15 | 19.42±3.2 |
| **Q** | 36.28±2.46 | 33.37±9.32 | 101.47±27.8 | 11.96±2.86 | 5.57±2.33 | 4.53±2.12 | 5.56±1.75 | 24.98±11.5 | 6.52±1.19 | 33.79±12.74 | 20.91±8.74 | 11.9±0.68 |
| **R** | 41.77±9.55 | 20.21±3.8 | 24.35±6.34 | 10.02±3.41 | 4.77±1.77 | 2.87±2.06 | 2.58±1.79 | 24.35±6.34 | 4.88±2.05 | 14±3.77 | 8.84±4.07 | 7.41±3.45 |
| **S** | 44.06±13.76 | 26.62±5.31 | 78.81±31.78 | 24.35±6.34 | 5.93±1.14 | 4.59±2.82 | 3.33±1.67 | 144.71±41.04 | 4.77±2.02 | 54.06±11.98 | 25.76±5.27 | 33.45±7.32 |
| **T** | 46.27±11.34 | 33.45±7.37 | 98.92±36.7 | 18.1±4.05 | 7.5±1.55 | 5.14±1.93 | 3.03±1.87 | 119.43±39.05 | 11.7±3.32 | 105.84±15.55 | 24.42±3.19 | 22.6±4.5 |
| **V** | 40.77±6.59 | 23.28±3.08 | 61.38±14.66 | 10±3.75 | 10.25±3.18 | 5.03±1.89 | 3.44±1.73 | 121.7±34.59 | 62.83±13.91 | 75.04±18.53 | 25.35±6.34 | 25.41±6.81 |
| **W** | 32.39±4.15 | 25.47±2.95 | 24.52±6.54 | 6.66±3.12 | 4.46±2.14 | 3.82±1.21 | 2.03±1.88 | 9.3±2.67 | 8.41±3.84 | 9.39±2.16 | 5.97±2.03 | 5.68±1.13 |
| **Y** | 31.23±0.54 | 52.65±4.23 | 52.64±8.14 | 11.07±6.3 | 5.88±3.02 | 5.67±0.82 | 3.91±2.79 | 38.44±2.9 | 12.1±4.48 | 14.09±3.83 | 11.9±2.42 | 10.38±2.03 |
