# Supplementary material for: Induction of hepatitis B core protein aggregation targeting an unconventional binding site
Source: eLife. 2025 Mar 26;13:RP98827. doi: 10.7554/eLife.98827 (PMC11942178; doi:10.7554/eLife.98827)
Supplement: Supplementary file 4. — Summary of cryo-EM data acquisition and image processing of the HBc CLPs with bound geraniol, P1dC, and SLLGRM-dimers. [file elife-98827-supp4.docx]

| **Data Acquisition and Image Processing** | | | | | | | |
| --- | --- | --- | --- | --- | --- | --- | --- |
|  | **HBc CLPs + Geraniol (2)** | | **HBc CLP+ SLLGRM dimer (4)** | | | **HBc CLP + P1dC**  **(7)** | |
| **Microscope** | Krios G3 300 kV, with Falcon III camera in linear mode | | | | | | |
| **Illumination** | Spot 5 nano probe; C2 aperture =70 µm; beam diameter 1 µm, | | | | | | |
| **Imaging** | Magnification: 75000 x, calibrated Pixel size: 1.064 Å/Px, objective aperture: 100 µm | | | | | | |
| **Exposure** | Total exposure = 40 e^-^/Å² in 20 fractions; exposure time: 2.6-3.2 s | | | | | | |
| **Targeted underfocus** | 600-1400 nm | | | | | | |
| **Movies per stage position** |  | 15 in 5 holes | | 15 in 5 holes | | | 3 in 1 hole |
| **Movies** | 4875 | | 4956 | | 2784 | | |
| **Template picked Particles** | 331635 | | 320845 | | 36817 | | |
| **Particles in final map** | 229646 | | 132610 | | 22830 | | |
| **Resolution Relion** | 2.8 Å* | | 2.8 Å* | | 3.6 Å | | |
| **Resolution CryoSparc** | n/a | | 2.5Å* | | 3.0 Å | | |
|  | **Models and Maps** | | | | | | |
| **MolProbity score** | 1.61 | | 1.58 | | 1.43 | | |
| **Clash score** | 4.79 | | 4.47 | | 6.2 | | |
| **Ramachandran Favored** | 94.7% | | 94.9% | | 97.6% | | |
| **Ramachandran Allowed** | 5.3 % | | 5.1% | | 2.4% | | |
| **Ramachandran Outliers** | 0 % | | 0% | | 0% | | |
| **Rotamer outliers** | 0% | | 0% | | 0% | | |
| **d FSC model (0/0.143/0.5)** | 2.5Å/2.6Å/2.9Å | | 2.1 Å /2.2 Å /2.7 Å | | 2.6Å/2.7Å/3.2Å | | |
| **Accession Codes** | PDB: 8PWO  EMD-17996 | | PDB: 8PX6  EMD-18001 | | PDB: 8PX3  EMD-18000 | | |
